# “Age is associated with Achilles tendon thickness in older adults: an ultrasound case–control study with intra-observer reliability”

**DOI:** 10.3389/fphys.2026.1821673

**Published:** 2026-05-13

**Authors:** Jorge Posada-Ordax, Marta Elena Losa-Iglesias, Ricardo Becerro de Bengoa-Vallejo, Eduardo Pérez-Boal, Bibiana Trevissón-Redondo, Israel Casado-Hernández, Antonio Javier Casanova-Malpica, Eva María Martínez-Jiménez, Hugo Espigares-Martínez, Francisco Javier Ruiz-Sánchez

**Affiliations:** 1 Department of Nursing and Physiotherapy, Universidad de León, León, Spain; 2 Department of Nursing and Stomatology, Universidad Rey Juan Carlos, Madrid, Spain; 3 Department of Nursing, Faculty of Nursing, Physiotherapy and Podiatry, Universidad Complutense de Madrid, Madrid, Spain; 4 FEBIO Research Group, Universidad Complutense de Madrid, Madrid, Spain; 5 Instituto de Investigación Biosanitaria de León (IBIOLEÓN), Universidad de León, León, Spain; 6 SALBIS Research Group, Universidad de León, León, Spain; 7 INSTIFEM, Universidad Complutense de Madrid, Madrid, Spain

**Keywords:** Achilles tendon, adult, obesity, reliability, tendon thickness, ultrasound

## Abstract

**Introduction:**

Obesity is associated with metabolic, inflammatory, and mechanical alterations that may affect the structure of the Achilles tendon, especially in older adults, who present increased tendon vulnerability due to age-related degenerative changes. To compare the ultrasound thickness of the Achilles tendon between sedentary older adults with obesity and those with normal weight, and to evaluate the intra-observer reliability of ultrasound measurements.

**Methods:**

A case–control study was conducted with 120 sedentary older adults (60 obese; 60 normal weight). The thickness of the right Achilles tendon was measured by ultrasound in the longitudinal plane at 4cm from the calcaneal insertion. Each participant was assessed using three consecutive measurements. Groups were compared using the independent samples t-test. Intra-observer reliability was analyzed using ICC (2,1).

**Results:**

The obese group showed a significantly greater tendon thickness compared with the control group (p = 0.003). However, the obese group was significantly younger than the control group. After adjusting for age in a linear regression model, age was the only significant factor associated with tendon thickness (p = 0.002), whereas obesity lost its significance (p = 0.143). Intra-observer reliability was excellent in both groups (ICC = 0.998 in controls; ICC = 0.986 in obese participants), with low SEM and MDC values.

**Discussion:**

Ultrasound measurement of Achilles tendon thickness demonstrated excellent intra-observer reliability in sedentary older adults. Although obese participants showed greater tendon thickness in the unadjusted analysis, age was significantly associated with tendon thickness after adjustment, suggesting that aging may play an important role in tendon structural characteristics in this population.

## 1 Introduction

Obesity is a chronic, non-communicable, and multisystemic disease, defined by an abnormal or excessive accumulation of body fat that may negatively affect health (Cheung et al., 2010). In 2016, it was estimated that 650 million adults worldwide were living with obesity, following an accelerated increase that began in the 1970s, which has consolidated this condition as a global public health epidemic (Gui et al., 2020). In 2024, it was estimated that more than 1 billion people were living with obesity, a figure that reflects a sustained upward trend over recent decades. Projections are even more concerning: by 2035, approximately 1.53 billion people, representing around 54% of the adult population, are expected to have obesity, while 1.77 billion will be overweight. In addition, it is estimated that 1 in every 8 deaths attributable to non-communicable diseases is related to excess body weight, highlighting the clinical and social impact of this condition (Simó et al., 2018). In Spain, the prevalence of obesity reaches 22%, with an additional 36.1% of the population being overweight, resulting in 58.1% of individuals with excess body weight. Abdominal obesity affects 64.7% of the population, reaching 91.6% among adults over 65 years of age (Simó et al., 2022).

Consequently, obesity is associated with a high number of comorbidities, particularly cardiometabolic diseases such as hypertension, dyslipidemia, type 2 diabetes, and coronary heart disease, as well as hepatic steatosis, obstructive sleep apnea, osteoarthritis, and mental health disorders. In addition, it increases the risk of heart failure, chronic kidney disease, and certain types of cancer, shortening life expectancy by approximately 3–4 years (Yang et al., 2022). Likewise, obesity is associated with significant musculoskeletal alterations, such as accelerated osteoarthritis, degenerative changes in the spine, increased lumbar lordosis, chronic low back pain, greater joint pressure in the lower limbs, relative muscle weakness, and gait alterations with increased energy expenditure. Furthermore, the chronic inflammatory state contributes to joint damage and loss of function (Carpi-Santos et al., 2022). Specifically, obesity is associated with an increased risk of tendinopathy, particularly at the level of the Achilles Tendon (AT), where a progressive increase in risk has been described according to the severity of obesity. This elevated risk appears to be mediated by both excessive mechanical loading and the chronic inflammatory environment and metabolic dysfunction characteristic of obesity (Bringmann et al., 2006). In this regard, recent human studies have demonstrated a causal link between obesity and Achilles tendinopathy, with a 44% increase in risk for each increase equivalent to one standard deviation in BMI. This effect is due to both mechanical overload (the AT can withstand up to eight times body weight during running) and metabolic and inflammatory mechanisms, including the release of pro-inflammatory adipokines, increased metalloproteinases, collagen glycation, and reduced TGF-β. These alterations promote extracellular matrix degradation, intratendinous edema, and reduced tendon repair capacity, predisposing to degeneration and injury (Miller et al., 2022).

Regarding diagnostic assessment, AT thickening is a characteristic ultrasound finding of tendinopathy, being more frequent in symptomatic patients and exceeding the normative range in approximately 73% of cases (Lin et al., 2025). Musculoskeletal ultrasound is a valid and effective tool for tendon assessment, with sensitivity and specificity comparable to magnetic resonance imaging in multiple studies. In addition, its ability to dynamically evaluate the tendon, its accessibility, and the additional information provided by elastography on tissue stiffness make it a reference technique for both diagnosis and early detection of degenerative changes (Huang et al., 2025). However, although current evidence has described the effects of obesity on tendons, a large proportion of studies have been conducted in athletic populations (Liu et al., 2015), laboratory animals (Vos et al., 2025), cell cultures (Corano Scheri et al., 2026), and biomechanical simulations (Deng et al., 2024), leaving a significant gap in knowledge regarding how excess body weight affects the AT in the general population and, especially, in older adults. This aspect is particularly relevant, given that aging itself is associated with degenerative tendon changes, such as decreased cellular density, collagen alterations, and reduced tissue repair capacity (Wang et al., 2022). Furthermore, in older adults, changes in the AT affect not only its morphology but also its function and stability during gait, which may have implications for fall risk. Recently, it has been shown that older adults present approximately 30% lower AT stiffness compared with younger adults, and that this reduced musculotendinous integrity is associated with alterations in stability control, reflecting greater vulnerability to imbalance and potential falls. In particular, lower plantar flexor strength in older adults was correlated with greater lateral instability, suggesting that the function of the triceps surae and the AT plays a relevant role in dynamic stability in this population (Becker et al., 2021).

It is well-established that musculoskeletal impairments associated with chronic loading often begin in early childhood. Recent research demonstrated that structural changes, such as increased meniscal stiffness, are already detectable in obese children and adolescents. These early alterations suggest that the cumulative effect of mechanical stress on weight-bearing tissues starts prematurely and becomes significantly more pronounced as individuals age (Sun et al., 2021). Consequently, investigating these effects in adult populations is crucial to understanding the long-term progression of tendon adaptations, such as those observed in the AT. In addition, Achilles tendinopathy affects approximately 2–3 per 1000 adults (Stuart et al., 2019). Lower-extremity tendinopathies have a reported prevalence of 16.6 per 1000 patients and an incidence of 7.9 per 1000, with Achilles tendinopathy representing one of the most frequent presentations (prevalence 5.2 per 1000; incidence 1.7 per 1000) (McGinnis et al., 2019). Beyond its frequency, Achilles tendinopathy has a substantial impact on health-related quality of life, particularly affecting mobility (66%), usual activities (50%) and pain/discomfort (89%). It also reduces work productivity in 38% of patients and leads to absenteeism in 9%. Furthermore, it imposes a considerable healthcare burden, with approximately nine healthcare visits per year and an estimated annual cost of €840 per patient, highlighting its significant clinical and socioeconomic impact (Skinnider et al., 2021). Furthermore, structural alterations in the Achilles tendon can be present in asymptomatic individuals and may precede the development of clinical symptoms. Furthermore, ultrasound-detected abnormalities have been associated with an increased risk of developing symptomatic tendinopathy, suggesting that early changes in tendon structure may represent preclinical stages of the condition (Hu et al., 2025; Lin et al., 2026).

In this context, the relationship between obesity and Achilles tendinopathy is well documented, as are age-related tendon morphological modifications (Qiu et al., 2017; Gulati et al., 2020) and the usefulness of ultrasound as a reliable assessment method (Finak et al., 2015). However, there is limited evidence simultaneously examining obesity, aging, and ultrasound AT thickness in older individuals without tendon symptoms, highlighting the need for specific investigations such as the present study.

Therefore, we pose the following research question: Is obesity in sedentary older adults associated with greater ultrasound AT thickness, and does this ultrasound measurement show an adequate degree of intra-observer reliability in this population?

Our hypothesis is: Obesity in older adults is associated with greater ultrasound AT thickness, and this ultrasound measurement demonstrates high intra-observer reliability. The aim of this study was to investigate the association between obesity, ageing, and Achilles tendon thickness in asymptomatic older adults, in order to better understand early structural tendon changes in the absence of clinical symptoms.

## 2 Materials and methods

### 2.1 Sample size

Sample size calculation was performed by t test family using correlation statistical tests using the G*Power 3.1.9.7 software version, with normal distribution, two-tailed hypothesis, a large effect size of 0.50, an α error probability of 0.05, with a β level of 20% and a desired power analysis of 80% (1-β error probability) were used for the sample size calculations. at least 26 participants must be studied. Therefore a total sample size of 120 participants was recruited. A sample were recruited by a consecutive sampling method using a successive and non-randomized simple method. Individuals were enlisted from the University of León Podiatry Clinic (Ponferrada - Spain).

The study was conducted following a prospective cross-sectional observational study design. The independent variables included age and obesity status, while the dependent variable was Achilles tendon thickness assessed by ultrasound. The study was carried out in full compliance with the Declaration of Helsinki and current regulations for research involving human individuals (Zhou et al., 2019). In addition, the recommendations of the “Strengthening the Reporting of Observational Studies in Epidemiology (STROBE)” initiative were followed (Wu et al., 2021; Szklarczyk et al., 2023). The Ethics Committee of the University of León (Castile and León, Spain) approved the research protocol (internal registration: ETICA-ULE-116-2025). Prior to participation, all individuals signed the corresponding informed consent.

Inclusion criteria comprised participants aged 65 years or older who were able to ambulate without assistance, had no history of AT surgery or active tendinopathy, and who, at the time of evaluation, did not report pain or stiffness in the AT.

Participants who met the minimum physical activity recommendations established by the World Health Organization (WHO) were excluded, namely those who accumulated between 150 and 300 minutes per week of moderate-intensity aerobic physical activity, between 75 and 150 minutes of vigorous-intensity activity, or an equivalent combination of both intensities. This criterion was applied to ensure that the sample consisted of individuals with insufficient or predominantly sedentary physical activity levels (Bader and Hogue, 2003; Otasek et al., 2019). Participants with active rheumatologic diseases, severe peripheral neuropathy, diabetes mellitus, recent treatment with tendon infiltrations, a history of ankle or AT surgery, systemic inflammatory disorders, with potential impact on the tendon were also excluded, as well as those who had sustained lower limb injuries within the previous 12 months that required immobilization. Individuals who had received fluoroquinolones in the previous 12 months were also excluded, as safety warnings from the FDA and the EMA indicate their association with the development of tendinitis and AT rupture even weeks or months after treatment (Chin et al., 2014; Morabito et al., 2023).

For sample selection according to BMI, the following classification was used: underweight (< 18.5 kg/m²), normal weight (18.5–24.9 kg/m²), overweight (25.0–29.9 kg/m²), class I obesity (30.0–34.9 kg/m²), class II obesity (35.0–39.9 kg/m²), and class III or morbid obesity (≥ 40.0 kg/m²). Participants included in this group met the BMI criteria corresponding to one of the obesity grades established by the WHO (Liu et al., 2008; Chen, 2015).

#### 2.1.1 Obese group

The obese group included 60 sedentary older adults with obesity, equally distributed between men and women. The mean age was 72.5 ± 6.4 years. Mean body weight was 89.6 ± 15.1 kg, and mean height was 163.1 ± 11.2 cm. The mean body mass index (BMI) was 33.5 ± 2.9 kg/m², confirming the classification of obesity according to the WHO criteria for body mass index.

#### 2.1.2 Control group

The control group included 60 sedentary, non-obese older adults, equally distributed between men and women. The mean age was 79.3 ± 7.8 years. Mean body weight was 65.3 ± 10.3 kg, and mean height was 161.1 ± 31.4 cm. The mean BMI was 23.5 ± 2.3 kg/m², corresponding to the normal weight range.

### 2.2 Equipment and materials

To perform the ultrasound measurements, a portable Mindray^®^ M6 laptop-type ultrasound system (Mindray Bio-Medical Electronics Co., Ltd., China; year of manufacture: 2012) was used. Image acquisition was carried out using a high-frequency linear transducer L14-6Ns, with an operating range between 6 and 14 MHz.

### 2.3 Study protocol

**Figure 1:**
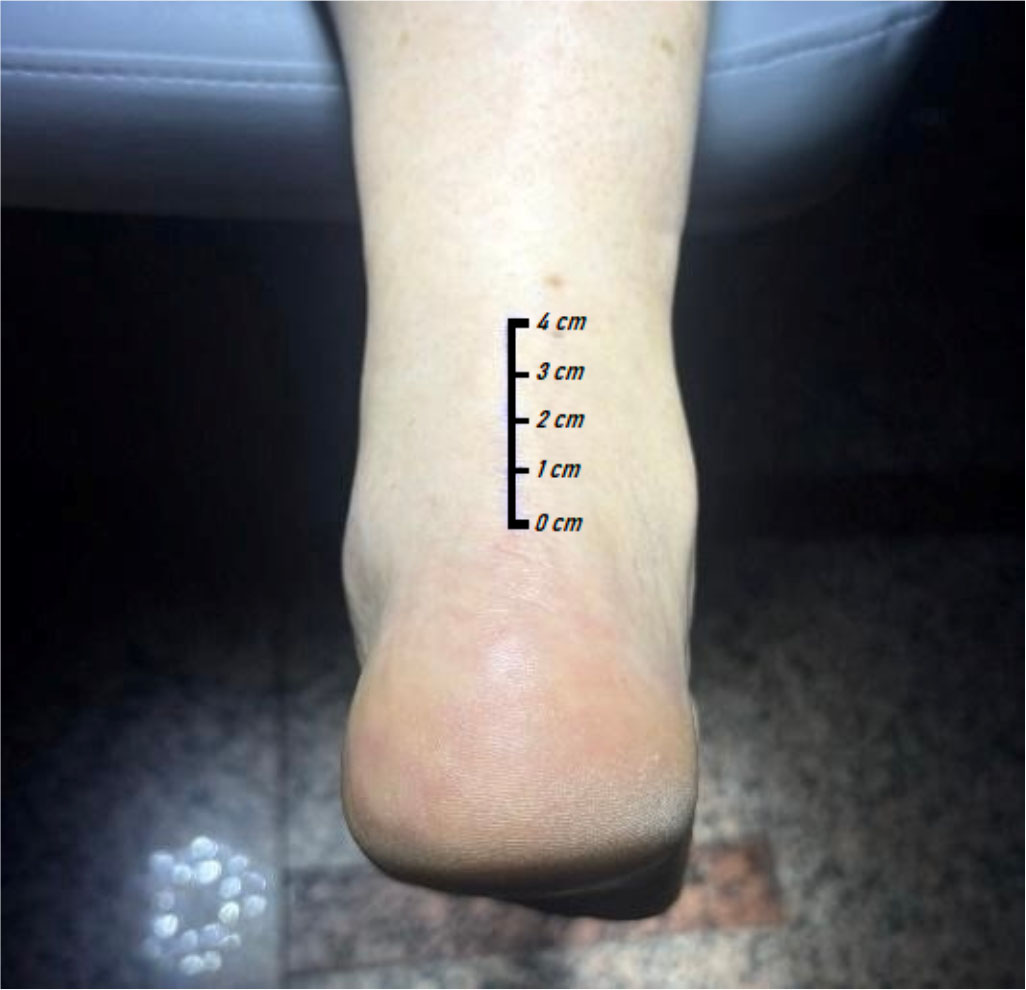
Ultrasound measurements performed 4cm proximal to the Achilles tendon insertion and at the mid-portion of the tendon.

For the ultrasound evaluation of the AT, a standardized protocol was followed. Participants were placed in the prone position, with the feet extending beyond the distal edge of the examination table to ensure relaxation of the triceps surae and the tendon. Measurements were performed exclusively on the right AT, in the longitudinal plane (**Figure 1**), at a fixed distance of 4 cm proximal to the calcaneal insertion, following recommendations described in the literature (Newman et al., 1984). To ensure consistency of the acquisition site, the measurement point was previously marked on the skin using a dermographic pen. Three independent ultrasound images were then acquired at this marked location, obtaining one measurement from each image. Three consecutive measurements of tendon thickness were obtained in each subject, taking care not to apply compression with the transducer on the skin or the tendon. The average of these three measurements was used as the representative value for statistical analysis. All examinations were performed by an ultrasonographer with 10 years of experience, ensuring consistency, standardization, and reliability of the measurements (Peña et al., 2024). Tendon thickness was defined as the anteroposterior (AP) diameter of the Achilles tendon, measured in the longitudinal plane at the selected point.

Ultrasound images were acquired using a standardized setting: gain was adjusted between 70 dB and 85 dB, the linear transducer frequency was set at 10 MHz, and a single focal zone was used, positioned at a depth of 0.5cm, corresponding to the location of the AT in order to optimize image resolution.

### 2.4 Statistical analysis

To analyze the intra-observer reliability of ultrasound measurements of the AT, the intraclass correlation coefficient (ICC 2,1) was used, obtained from three consecutive measurements, together with its 95% confidence interval (95% CI) as an indicator of precision. In addition, the coefficient of variation (CV), the standard error of measurement (SEM), and the minimal detectable change (MDC) were calculated in order to quantify absolute error and determine the minimum magnitude of change not attributable to measurement variability.

For interpretation of these values, the categories established by Landis and Koch (Wang et al., 2024) were used as a reference, where values equal to or less than 0.20 are considered poor, values between 0.21 and 0.40 fair, between 0.41 and 0.60 moderate, between 0.61 and 0.80 substantial, and greater than 0.81 almost perfect. In addition, following the recommendations of Portney and Watkins (Ben et al., 2024), clinical measurements presenting an ICC greater than 0.90 were considered reliable, as this increases the likelihood that results are consistent.

Data analysis was performed using SPSS software for Windows (version 22.0; SPSS Inc., Chicago, IL, USA). Data distribution normality was assessed using the Shapiro–Wilk test and showed approximate normal distribution. Quantitative variables were described using the mean and standard deviation (SD), accompanied by the 95% confidence interval.

To compare demographic characteristics and ultrasound measurements of AT thickness between the obese and control groups, the independent samples t-test was used. The level of statistical significance was set at p < 0.05. Additionally, a linear regression analysis was performed to evaluate the association between obesity and Achilles tendon thickness after adjusting for age.

## 3 Results

Regarding sociodemographic characteristics, statistically significant differences were observed between the control and obese groups in age, body weight, and BMI (p < 0.001 in all cases), with the obese group being younger and presenting higher body weight and BMI values. No significant differences were found in height between the two groups (p > 0.05) (**Table 1**).

**Table 1:** Sociodemographic characteristics.

Variable	Control group Mean ± SD (IC95%)	Obese group Mean ± SD (IC95%)	Total Mean ± SD (IC95%)	P value (T student test)
Age (years)	79.3 ± 7.8 (77.3 – 81.3)	72.5 ± 6.4 (70.8 – 74.1)	75.9 ± 7.9 (74.5 – 77.3)	P < 0.001
Body Mass (kg)	65.3 ± 10.3 (62.7 – 68)	89.6 ± 15.1 (85.7 – 93.5)	77.5 ± 17.7 (74.3 – 80.6)	P < 0.001
Height (cm)	161.1 ± 31.4 (153 – 169.2)	163.1 ± 11.2 (160.2 – 166)	162.1 ± 23.5 (157.9 – 166.3)	P = 0.641
BMI (kg/m^2^)	23.5 ± 2.3 (22.9 – 24.1)	33.5 ± 2.9 (32.8 – 34.2)	28.5 ± 5.7 (27.5 – 29.5)	P < 0.001

The values are presented as mean ± standard deviation (SD) and 95% confidence interval (95% CI). Comparisons between the control group and the obese group were performed using the independent samples T Student test. BMI, body mass index.

**Figure 2:**
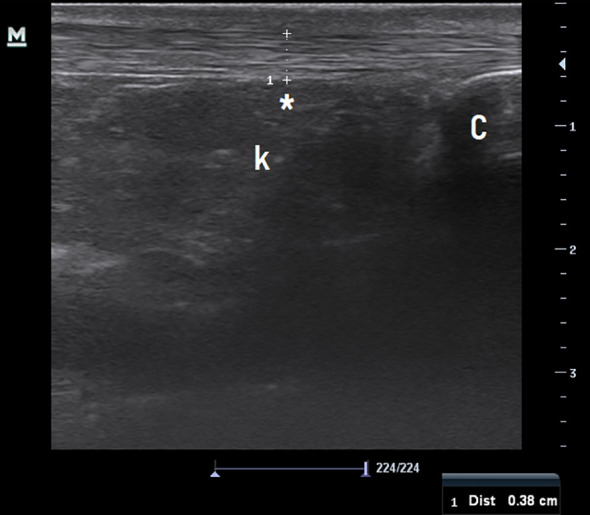
Ultrasound measurement of the Achilles tendon in a normal-weight participant (control group). Asterisk: Achilles tendon thickness at the midportion; C: Calcaneus. K: Kager’s fat pad.

With respect to AT thickness, analysis using independent samples t-test revealed a significant difference between groups, with the obese group showing significantly greater tendon thickness compared with the control group (**Figure 2**) (p = 0.003) (**Table 2**).

**Table 2:** Linear regression analysis evaluating the association between obesity, age, and Achilles tendon thickness.

Variable	β coefficient	P value
Obesity	0.027	P = 0.143
Age (years)	−0.003	P = 0.002

Linear regression model evaluating the association between obesity and Achilles tendon thickness adjusted for age. **β:** regression coefficient. P value for the regression coefficient.

However, to account for the significant age difference between groups, a linear regression analysis adjusted for age was performed. The results showed that age was significantly associated with Achilles tendon thickness (β = −0.003, p = 0.002), whereas obesity was not significantly associated after adjustment (β = 0.027, p = 0.143) (**Table 3**).

**Table 3:** Comparison of Achilles tendon thickness between obese and control groups.

Variable	Control group Mean ± DS (IC95%)	Obese group Mean ± DS (IC95%)	Total Mean ± DS (IC95%)	P value (T student test)
Tendon thickness	0.5 ± 0.1 (0.5 – 0.6)	0.6 ± 0.1 (0.6 – 0.6)	0.6 ± 0.1 (0.5 – 0.6)	P = 0.003

The values are presented as mean ± standard deviation (SD) and 95% confidence interval (95% CI). Comparisons between the control group and the obese group were performed using the independent samples T Student test.

Intra-observer reliability of ultrasound measurements was excellent in both groups. ICC (2,1) showed values greater than 0.98 in the control group and greater than 0.97 in the obese group, which, according to the commonly used classification (ICC > 0.90 = excellent reliability), confirms very high consistency. SEM and MDC values were low in both groups, indicating minimal variability attributable to the measurement procedure (**Table 4**).

**Table 4:** Intra-observer reliability of Achilles tendon thickness measurements in both groups.

Variable	Mean (SD)	IC95%	CV (%)	ICC (2.1) (IC95%)	SEM	MDC	95% normality values
Control Group	0.5 (0.1)	0.5 – 0.6	15.5	0.99 (0.99 – 0.99)	0.03	0.09	0.4 – 0.7
Obese Group	0.6 (0.1)	0.6 – 0.6	18	0.98 (0.97 – 0.99)	0.01	0.03	0.4 – 0.8

The mean, standard deviation (SD), 95% confidence interval (95% CI), CV, coefficient of variation, intraclass correlation coefficient [ICC(2,1)] with its 95% CI, SEM, standard error of measurement; MDC, minimal detectable change; and 95% normality values are reported.

## 4 Discussion

Although the obese group showed a statistically significant increase in Achilles tendon thickness compared with the control group in the unadjusted analysis, the absolute difference between groups was relatively small. Furthermore, the linear regression analysis performed in the present study showed that age was significantly associated with Achilles tendon thickness, whereas obesity was not significantly associated after adjustment for age. Therefore, the clinical relevance of the observed differences should be interpreted with caution. These findings suggest that the increase in tendon thickness initially observed in obese individuals may be at least partially explained by age-related structural changes, as our regression model confirmed that aging, rather than excess body weight, is the primary driver of these morphological changes. However, according to recently published normative values, Achilles tendon thickness in the midportion region in asymptomatic individuals shows a median of 4.9 mm (95% reference interval: 3.8–6.9 mm), while in the insertional region values are approximately 3.7 mm (2.8–4.8 mm). When comparing these values with the results of our study, tendon thickness in both groups falls within the normative ranges reported in the literature (Lin et al., 2025). Therefore, although statistically significant differences were observed between groups, the magnitude of this difference appears to be limited from a clinical perspective and may reflect subtle structural variations rather than clinically relevant pathological changes.

The present study showed that sedentary older adults with obesity exhibited greater AT thickness than normal-weight individuals. However, the regression analysis indicated that age was significantly associated with tendon thickness, whereas obesity was not significantly associated after adjusting for age. In addition, ultrasound measurements demonstrated excellent intra-observer reliability, reinforcing the validity of the protocol used and ensuring that the differences observed between groups reflect true changes rather than technical variability. These findings are particularly relevant in the elderly population, given the functional role of the AT in stability and gait, as well as the potential added risk of tendon degeneration associated with obesity and senescence proposed by previous studies, which identify tissue aging, chronic inflammation related to obesity, and sarcopenia as factors contributing to tendon degeneration (Sakami et al., 2019).

Previous evidence has consistently shown that excess body weight has a negative impact on AT morphology and mechanics. A study conducted in young and middle-aged physically active men without tendon pathology showed that obesity is associated with greater AT thickness and an altered mechanical response after exercise (Cayrol and Girard, 2014). In the present study, sedentary older adults with obesity exhibited greater tendon thickness than the normal-weight group in the unadjusted analysis. While the previous study analyzed the immediate adaptation of the tendon to exercise, our results suggest that these morphological differences may also be present at rest in older adults. Taken together, while previous literature highlights the impact of excess body weight on tendon morphology, our regression analysis demonstrated that age is the predominant factor associated with increased tendon thickness in this population. This suggests that the natural process of aging may have a greater influence than obesity on tendon structural changes in sedentary older adults.

From the perspective of physical activity, a study in recreational runners analyzed the impact of adiposity on AT morphology and observed that in young normal-weight individuals, regular running practice was associated with greater tendon thickness, interpreted as a physiological hypertrophy induced by mechanical loading (Mengge et al., 2025). However, when overweight was present, both runners and sedentary individuals showed tendon thickening accompanied by a higher frequency of ultrasound abnormalities and neovascularization, suggesting that in the presence of adiposity, increased thickness may reflect a potentially pathological rather than adaptive phenomenon. In contrast to this pattern observed in young populations, our unadjusted results showed greater AT thickness in sedentary older adults with obesity compared with normal-weight individuals, although the regression analysis indicated that age was significantly associated with tendon thickness. Taken together, our findings highlight that the structural alterations of the AT observed in this sample are fundamentally associated with the aging process. Our study extends existing knowledge by demonstrating that in sedentary older adults, age acts as a stronger driver of tendon thickening than excess body weight, emphasizing the profound impact of senescence on tendon vulnerability.

When analyzing the effects of age on the tendon, aging entails a series of progressive changes that significantly affect tendon structure and function, reducing its capacity to maintain homeostasis and respond adequately to mechanical loads. From a biomechanical standpoint, aged tendons exhibit lower tensile strength, alterations in elastic modulus, and reduced viscoelastic properties, which decrease their ability to tolerate repetitive loads and increase susceptibility to microtears and tendinopathy. In addition, the healing process is compromised due to a chronic pro-inflammatory state (“inflammaging”), repair based on a higher proportion of type III collagen and scar tissue, leading to slower and lower-quality regeneration. Together, these mechanisms explain why advanced age constitutes a determining factor in tendon vulnerability and in the appearance of structural alterations even in the absence of clinical symptoms (Santofimia-Castaño et al., 2018).

When specifically addressing the reliability of ultrasound measurements of AT thickness, previous studies evaluating twenty adults by measuring tendon thickness at 4cm and 6 cm from the calcaneal insertion reported very high reliability values, with an ICC of 0.997 for fixed acquisition at rest, based on measurements performed by two examiners (Newman et al., 1984). In another investigation conducted with one hundred participants, fifty with a clinical diagnosis of Achilles tendinopathy and fifty asymptomatic, intra- and inter-rater reliability was analyzed, showing excellent reliability in the midportion of the tendon for both intra-rater (ICC 0.98) and inter-rater (ICC 0.97) assessments (Makita et al., 2021). Similarly, in a study including fourteen adults without a history of tendinopathy or tendon pain, two sonographers performed the measurements, demonstrating very high intra-rater reliability (ICC > 0.98) and very low SEM and MDC values, confirming the high precision of the procedure (Ko et al., 2015). Finally, a systematic review analyzing 21 previous studies on ultrasound measurements of the AT, in both healthy individuals and those with tendinopathy, concluded that ultrasound techniques used to quantify tendon dimensions show intra- and inter-rater reliability ranging from good to excellent, depending on the protocol applied and the degree of standardization (Fields and Ghorpade, 2012). Compared with these previous studies, our work provides concordant results regarding the high reliability of ultrasound measurements of the AT, particularly in a sedentary elderly population. Moreover, although previously reported ICC values range from high to excellent, our results reinforce the robustness of ultrasound as a reliable tool for structural assessment of the AT.

Several limitations of this study should be considered. First and most importantly, the significant age difference between the obese and control groups acted as a confounding factor in the unadjusted analysis. Although we addressed this using an age-adjusted linear regression, future studies should use age-matched cohorts. Second, ultrasound measurements were only performed in the longitudinal axis and at rest, limiting the extrapolation of results to transverse sections or dynamic functional conditions. Third, inter-observer and inter-session reliability were not evaluated. Fourth, adiposity was assessed solely using BMI, without analyzing body fat distribution, which could differentially affect tendon structure. Another methodological limitation is determining the measurement point by marking the skin. Although we replicated a highly reliable protocol (ICC 0.998) (Newman et al., 1984), variations in subcutaneous tissue thickness, especially in individuals with obesity, can cause the surface mark to project onto slightly different tendon segments. To avoid this bias, future studies should prioritize ultrasound-guided internal anatomical landmarks or advanced modalities like shear wave elastography, which is highly effective for evaluating the Achilles tendon (Ndoja et al., 2020). Furthermore, using this surface mark and maintaining the same participant position during all acquisitions may have increased measurement consistency, potentially overestimating the observed intra-observer reliability. Fifth, only the right Achilles tendon was evaluated to standardize the protocol and optimize examination time, following the approach of previous studies (Newman et al., 1984). Consequently, potential differences related to limb dominance or bilateral variability were not accounted for.

As future lines of research, we propose to further investigate the reliability of ultrasound measurements of the AT by incorporating inter-observer and inter-session assessments to determine measurement stability when different examiners are involved and at different acquisition times. It is also necessary to analyze the reliability of measurements under load and during dynamic tasks, given that tendon mechanical behavior may change substantially under functional conditions and these measurements could provide more representative information about its real performance. Another line of interest is to examine AT thickness in physically active older adults, which would allow differentiation between the effects of aging and those derived from physical activity and exploration of potential beneficial adaptations compared with sedentary behavior. Additionally, should consider bilateral assessments to provide a more comprehensive understanding of tendon morphology and the effects of leg dominance. Finally, future research should systematically address intratendinous structural alterations, such as angiogenesis and changes in fibrillar pattern, using advanced ultrasound techniques or complementary methods, in order to improve understanding of early degenerative processes and their relationship with the morphological parameters evaluated.

In summary, while our initial analysis showed that sedentary older adults with obesity exhibited greater AT thickness even in the absence of symptoms, our regression model revealed that age appears to be the main factor associated with these basal structural changes. This result expands existing evidence by demonstrating that senescence, rather than excess body weight, exerts the most significant impact on tendon morphology in this population. This highlights the importance of considering aging as a critical factor in clinical assessment and in the design of future research aimed at the prevention and early detection of tendon alterations.

## 5 Conclusions

As final conclusions of this study, the reliability of ultrasound measurements of the AT in sedentary older adults, both obese and non-obese, was found to be high, supporting the use of ultrasound as a valid and reproducible tool for morphological assessment of the tendon in this population. In addition, age showed a significant association with Achilles tendon thickness, suggesting that aging may play an important role in tendon thickening in older adults.

## Data Availability

The raw data supporting the conclusions of this article will be made available by the authors, without undue reservation.
